# Circulating Vitamin D Levels and Risk of Vitiligo: Evidence From Meta-Analysis and Two-Sample Mendelian Randomization

**DOI:** 10.3389/fnut.2021.782270

**Published:** 2021-12-22

**Authors:** Jie Song, Ke Liu, Weiwei Chen, Bin Liu, Hong Yang, Linshuoshuo Lv, Xiaohui Sun, Yingying Mao, Ding Ye

**Affiliations:** Department of Epidemiology, School of Public Health, Zhejiang Chinese Medical University, Hangzhou, China

**Keywords:** 25-hydroxyvitamin D, 25-hydroxyvitamin D_3_, Mendelian randomization, meta-analysis, vitiligo

## Abstract

**Background:** The association between circulating vitamin D levels and risk of vitiligo was inconsistent among observational studies, and whether these observed associations were causal remained unclear. Therefore, we aimed to evaluate the effect of vitamin D on the risk of vitiigo using meta-analysis and Mendelian randomization (MR).

**Methods:** At the meta-analysis stage, literature search was performed in PubMed and Web of Science to identify eligible observational studies examining the association of circulating 25-hydroxyvitamin D [25(OH)D] or 25-hydroxyvitamin D_3_ [25(OH)D_3_] levels with risk of vitiligo up to April 30, 2021. Standardized mean differences (SMDs) with 95% confidence intervals (CIs) of 25(OH)D and 25(OH)D_3_ in patients with vitiligo relative to controls were pooled. Then at the MR stage, genetic instruments for circulating 25(OH)D (*N* = 120,618) and 25(OH)D_3_ (*N* = 40,562) levels were selected from a meta-analysis of genome-wide association studies (GWAS) of European descent, and summary statistics of vitiligo were obtained from a meta-analysis of three GWASs including 4,680 cases and 39,586 controls. We used inverse-variance weighted (IVW) as main method, followed by weighted-median and likelihood-based methods. Pleiotropic and outlier variants were assessed by MR-Egger regression and MR Pleiotropy RESidual Sum and Outlier (MR-PRESSO) test.

**Results:** In the meta-analysis, patients with vitiligo had a lower level of circulating 25(OH)D compared with controls [SMD = −1.40; 95% confidence interval (CI): −1.91, −0.89; *P* < 0.001], while no statistically significant difference of 25(OH)D_3_ between vitiligo cases and controls was found (SMD = −0.63; 95% CI: −1.29, 0.04; *P* = 0.064). However, in the MR analyses, genetically predicted 25(OH)D [odds ratio (OR) = 0.93, 95% CI = 0.66–1.31, *P* = 0.66] and 25(OH)D_3_ levels (OR = 0.95, 95% CI = 0.80–1.14, *P* = 0.60) had null associations with risk of vitiligo using the IVW method. Sensitivity analyses using alternative MR methods and instrumental variables (IV) sets obtained consistent results, and no evidence of pleiotropy or outliers was observed.

**Conclusion:** Our study provided no convincing evidence for a causal effect of 25(OH)D or 25(OH)D_3_ levels on the risk of vitiligo. Further longitudinal and experimental studies, as well as functional studies are warranted to elucidate the role of vitamin D in the development of vitiligo.

## Introduction

Vitiligo is an acquired pigmented skin disease caused by the loss of melanocyte function, which is characterized by localized or generalized complete depigmentation of skin mucosa. It can occur in all parts of the body, commonly in finger back, wrist, forearm, face, neck and around genitalia, affecting both men and women (1). Globally, the prevalence of vitiligo was estimated to be ranging from a low of 0.5% to a high of 2.0% in adults, and the peak onset period is between 10 and 30 years of age (2). Although vitiligo does not bring fatal risk, it is often accompanied by a variety of autoimmune system-related diseases, such as Hashimoto's thyroiditis, diabetes, Addison's disease and so on (3–6). Therefore, it is necessary to further explore the influence of modifiable risk factors on the incidence of vitiligo, which may provide new ideas for its prevention and treatment.

Previous studies have identified a series of nutritional factors associated with vitiligo, such as selenium (7), copper, and zinc (8). Recently, vitamin D deficiency has been reported to be a potential risk factor for vitiligo (9). With usual physiological intake, the majority of vitamin D is converted to 25-hydroxyvitamin D (25(OH)D) and released into the blood, thus circulating 25(OH)D concentrations can reflect vitamin D status in the human body. As the main metabolite of 25(OH)D, the levels of circulating 25-hydroxy vitamin D_3_ [25(OH)D_3_] is also widely accepted as an indicator of overall vitamin D status (10). Though no randomized controlled trials have been conducted to investigate the preventive role of vitamin D on the risk of vitiligo to date (11), numerous observational epidemiological studies have evaluated circulating 25(OH)D and 25(OH)D_3_ levels in patients with vitiligo relative to controls. For instance, Atazadeh et al. (12) conducted a case-control study with 90 vitiligo patients and 90 healthy controls in Iran, and found that the median (interquartile range) of circulating 25(OH)D level was lower in patients with vitiligo than in matched healthy controls [28.50 (22.52–33.20) ng/mL vs. 29.10 (27.40–35.70) ng/mL, *P* = 0.01). Another case-control study (13) conducted in India with 100 vitiligo patients and 100 healthy controls suggested that circulating 25(OH)D_3_ levels were lower in vitiligo patients than controls (16.17 ± 8.63 vs. 25.49 ± 1.02 ng/ml, *P* < 0.001). However, Saniee (14) recruited 98 patients with vitiligo and 98 matched healthy controls and measured 25(OH)D levels, but did not detect statistically significant differences between patients with vitiligo and normal subjects (22.37 ± 10.78 vs. 26.16 ± 17.11 ng/ml, *P* = 0.19). Similarly, Ustun et al. also found no statistically significant association (15). These inconsistent results may be due to differences in the study population, sample size, measurement methods and so on. Besides, findings from traditional observational studies are susceptible to bias such as confounding and reverse causation, therefore, it remains unclear whether the observed association was causal or not.

Mendelian Randomization (MR) study utilizes genetic variants such as single nucleotide polymorphisms (SNPs), which act as instrumental variables (IVs) to estimate the potential causal relationship between exposure and outcome (16). Since genotypes are randomly distributed in the process of gamete formation, the relationship between exposure and outcome of interest will not be biased by common confounding factors, such as postnatal environment, socio-economic status and behavioral factors in conventional observational studies. Moreover, MR study has unique advantages in causal inference because genetic variation is inherited from parents and remains unchanged after birth, so the association between genetic variation and outcome is reasonable in time sequence.

Therefore, in the present study, we first performed a systematic review and meta-analysis of existing observational evidence on the association of circulating 25(OH)D or 25(OH)D_3_ levels with risk of vitiligo, and then performed MR analysis to further investigate whether there was evidence for a causal relationship of circulating 25(OH)D and 25(OH)D_3_ levels with risk of vitiligo.

## Method

### Meta-Analysis

#### Data Source

**Figure 1:**
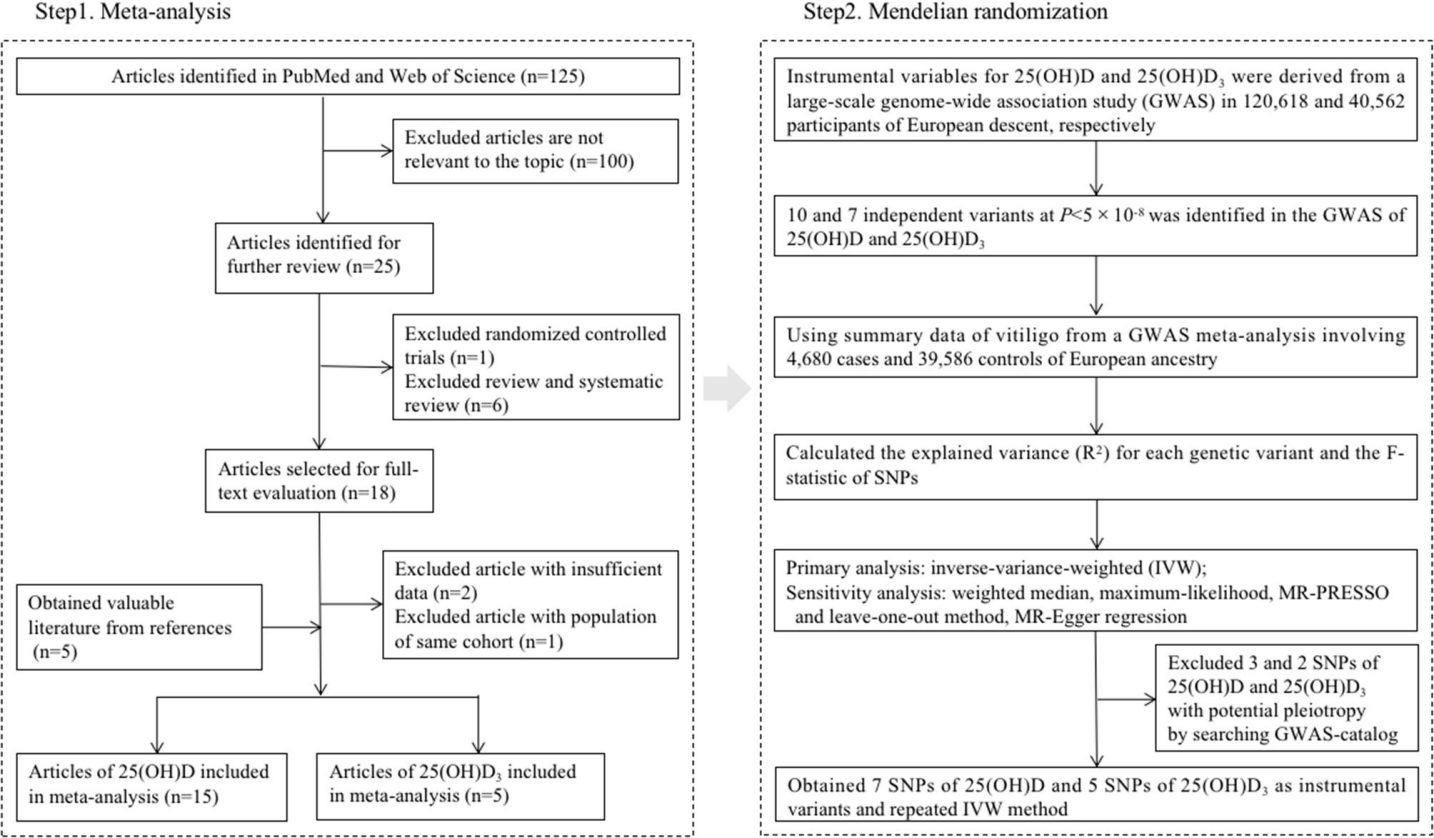
An overall design of the present study.

The overall design of the present study is shown in Figure 1. We first systematically searched PubMed and Web of Science up to April 30, 2021 to identify observational studies examining the association of circulating 25(OH)D or 25(OH)D_3_ levels with risk of vitiligo. The keywords included vitiligo AND (vitamin D OR 25-hydroxyvitamin D OR 25-dihydroxyvitamin D_3_ OR 25(OH)D OR 25(OH)D_3_ OR calcidiol).

#### Literature Inclusion and Exclusion Criteria

The inclusion criteria were: (i) The study was cross-sectional, case-control or cohort design; (ii) The original study reported standardized mean difference (SMD) and 95% confidence interval (95% CI) of circulating 25-hydroxyvitamin D or 25-hydroxyvitamin D_3_ between patients with vitiligo and controls, or provided sufficient data to calculate SMD and 95% CI; (iii) When two or more literatures recruited overlapped study population, the literature with the largest sample size was included. The exclusion criteria were: (i) Review or meta-analysis; (ii) Full text is unavailable; (iii) Data provided in the literature were insufficient.

#### Data Extraction and Quality Assessment

Relevant information was extracted and crosschecked by two researchers independently (Liu K and Chen W), and discussed with the third researcher (Ye D) in case of disagreement. The extracted information included the first author, year of publication, region, type of study design, study period, sample size, gender and age of the study participants. Two researchers (Liu K and Chen W) independently scored the quality of the included articles using Newcastle Ottawa scale (NOS) (17). The scores ranging from 7 to 9 were regarded as high quality, 5–6 as moderate quality, and ≤4 as low quality.

#### Statistical Analysis

The mean and standard deviations (SD) of circulating levels of 25 (OH) D and 25 (OH) D_3_ between patients with vitiligo and controls were used to calculate SMDs and 95% CIs. If the study provided with median and range, we computed mean and SD using previously described formulas (18, 19). If odds ratio (OR) was provided, we computed SMD using the formulae of SMD= ${\left(\frac{\sqrt{3}}{\pi }\right)}^{*}lnOR$ (20). The heterogeneity among studies was tested by Cochrane's Q test and *I*^2^ statistics. If *I*^2^ < 50% and *P* > 0.10, a fixed-effects model was used; otherwise, a random-effects model was applied. Sensitivity analysis was performed to evaluate the stability of the association by sequential removal of each study from the analysis. Subgroup analysis was performed in terms of region, publication year, quality score and study design, and meta-regression analysis was conducted for the above factors, respectively. Funnel plot was generated, and Begg's test (21) and Egger's test (22) were used to evaluate potential publication bias.

All statistical analyses were performed using STATA (version 14.0). Two-sided *P*-values < 0.05 were considered statistically significant.

### Mendelian Randomization

#### Outcome Dataset

Genetic association data of vitiligo were obtained from a meta-analysis of three genome-wide association studies (GWAS) involving 4,680 vitiligo cases and 39,586 controls of European ancestry (23). A total of 48 genetic variants associated with risk of vitiligo were identified, accounting for 17.4% of heritability. Detailed information of the above studies has been described in the previous article (23). All study participants provided written informed consent and the study was carried out under the jurisdiction of each local institutional review board.

#### Selection of Genetic Variants

IVs for 25(OH)D and 25(OH)D_3_ were derived from a large-scale GWAS including 120,618 and 40,562 participants of European descent, respectively (24). A total of ten and seven independent SNPs (*r*^2^ threshold < 0.1) associated with circulating 25(OH)D and 25(OH)D_3_ levels at genome-wide significance level (*P* < 5 × 10^−8^) were identified, respectively. None of the SNPs overlapped or were in linkage disequilibrium (*r*^2^ < 0.1) with known risk loci of vitiligo. The variance explained for total 25(OH)D by the ten lead SNPs was 3.95%, and the variance explained for 25(OH)D_3_ by the seven SNPs was 4.58%. The details of GWAS studies and datasets used in the present study are listed in Supplementary Table 1.

#### Statistical Analysis

We calculated F-statistics to quantify the strength of the IVs, with the equation of *F* = *R*^2^ × (*n* – 2)/(1 – R^2^), in which *R*^2^ represents the variance explained by the IVs and *n* indicates the sample size (25). We used inverse-variance weighted (IVW) method to evaluate the potential causal relationship of circulating 25(OH)D and 25(OH)D_3_ levels with risk of vitiligo as the main analysis. IVW method combines the causal effect estimates of multiple single genetic IVs by Wald ratio method (26). The estimated effect size by IVW method is essentially the regression coefficient of weighted regression without considering the intercept term (27). Moreover, weighted-median and likelihood-based methods were used to evaluate the robustness of the IVW method. For the weighted median method, the consistent estimation of the causal effect can be obtained as long as the weight of the causal effect calculated by the effective IV reaches 50% (25). Moreover, the likelihood-based method was used to evaluate the casual relationship under the assumption of a linear association between the risk factor and the outcome variables (28).

In addition, we conducted MR-Egger regression analysis to analyze the effect of potential pleiotropy on causal estimation. The intercept term of MR-Egger represents the average pleiotropic effect of genetic variation. If the intercept is different from zero, it indicates that there is evidence of directed pleiotropy (29). Also, we performed Mendelian randomization pleiotropy residual sum and outlier (MR-PRESSO) method (30) to detect and correct for horizontal pleiotropic outliers. It conducts a global test of heterogeneity by regressing the SNP-outcome association on the SNP-exposure association and comparing the observed distance of each SNP from the regression with that expected under the null hypothesis of no pleiotropy.

We further performed “leave-one-out” method as sensitivity analyses to test the reliability of the association of genetically predicted circulating 25(OH)D and 25(OH)D_3_ with risk of vitiligo. Specifically, we removed each SNP and combined the effect estimates of the remaining SNPs using the IVW method. The fluctuation of the results before and after removing the SNP reflects the stability of the association. Finally, considering the influence of pleiotropy on the results, associated phenotypes of the SNPs used as IVs were manually scanned through the GWAS Catalog (https://www.ebi.ac.uk/gwas, accessed on May 15, 2021). SNPs associated with other traits at genome-wide significance were excluded from the IVs and MR analyses were subsequently performed using the remaining SNPs.

R (version 4.0.5) with packages “MendelianRandomization” and “MR-PRESSO” was used for MR analysis. *P*-values < 0.05 were considered statistically significant.

## Results

### Patients With Vitiligo Had a Lower Level of 25(OH)D Relative to Controls

The flowchart of literature inclusion and exclusion is shown in Figure 1. Briefly, a total of 125 articles were retrieved in the initial stage, with 15 articles and 5 articles investigating the association of 25(OH)D (12, 14, 31–43) and 25(OH)D_3_ (13, 15, 44–46) with risk of vitiligo included in the final analysis, respectively. In total, we collected the data of 987 patients and 770 controls for 25(OH)D, and 305 patients and 321 controls for 25(OH)D_3_ between 2012 and 2020 in this meta-analysis. Overall, there were 14 case-control studies and one cross-sectional study for 25(OH)D, and 4 case-control studies and one cross-sectional study for 25(OH)D_3_. As assessed by NOS, the quality of the included studies was high in general. The basic information of the literature included is shown in Table 1.

**Table 1:** Characteristics of individual studies included in the meta-analysis.

**References**	**Region**	**Study type**	**Research time**	**Sample size**	**Sex, male (%)**		**Age (years)**		**NOS**
					**Cases**	**Controls**	**Cases**	**Controls**	
**25(OH)D**									
Xu et al. (39)	China	Case-control	Mar–May 2011	221	46.7	42	18–60	18–60	7
			Sep–Oct 2010	50			18–60	18–60	
Saleh et al. (35)	Egypt	Case-control	Apr–Jun 2011	80	45	45	34.1 ± 11.4	34.2 ± 11.5	8
Aksu Cerman et al. (31)	Turkey	Cross-sectional	Nov 2012–Mar 2013	102	23.9	29.1	33.64 ± 11.51	32.55 ± 9.78	7
Sehrawat et al. (36)	India	Case-control		60	33.3	33.3	31.33 ± 7.33	31.33 ± 7.33	8
Takci et al. (38)	Turkey	Case-control	Nov 2011–Feb 2012	87	54.5	23.2	34.5 ± 16.1	33.0 ± 12.6	7
Doss et al. (43)	Egypt	Case-control	Jun–Sep 2013	60	66.7	53.3	32.5 ± 14.6	28 ± 5.7	7
Sobeih et al. (37)	Egypt	Case-control	July–December 2013	150			31.5 ± 13.5	31.5 ± 13.5	7
Shalaby et al. (41)	Egypt	Case-control	Dec 2014–Jun 2016	80	75	62.5	28.70 ± 13.44	32.95 ± 9.24	7
Farag et al. (32)	Egypt	Case-control	Mar–Jun 2016	75	36	40	28.76 ± 10.64	28.16 ± 9.84	8
Ibrahim et al. (33)	Egypt	Case-control	Dec 2015–Dec 2016	100	50	50	34.27 ± 11.741	35.20 ± 10.65	7
Omidian and Asadian (34)	Iran	Case-control	Apr 2015–Mar 2016	60	40	36.6	36.93 ± 14.5	32.03 ± 15.08	7
Zhang et al. (40)	China	Case-control	Nov 2016–Mar 2017	214	43.9	47	7.37 ± 3.78	6.95 ± 3.63	7
Amer et al. (42)	Egypt	Case-control	Mar–Oct 2018	42	47.6	47.6	30.8 ± 19.1	30.6 ± 13.2	8
Saniee (14)	Iran	Case-control	Spring–summer 2017	196	51	54.1	30.06 ± 16.18	29.45 ± 13.16	7
Atazadeh et al. (12)	Iran	Case-control	2018–2019	180	62.2	62.2	18–65	19–60	7
**25(OH)D**_**3**_									
Ustun et al. (15)	Turkey	Cross-sectional	2010–2011	66	52	48.7	33.9 ± 19.4	34.7 ± 15.9	5
Abou Khodair et al. (46)	Egypt	Case-control	Mar–Jun 2018	60	20	33.3	30.7 ± 9.3	30.3 ± 9.7	7
Dinachandran and Pillai (45)	India	Case-control	Jan–Apr 2018	100	54	60	30.96 ± 10.57	31.45 ± 8.33	8
Alshiyab et al. (44)	Jordan	Case-control	May–Dec 2018	200	42	35	2–82	2–74	8
Hassan et al. (13)	India	Case-control	Mar–Apr 2019	200	39	40	28.66 ± 11.98	28.66 ± 11.98	7

*25(OH)D, 25-hydroxyvitamin D; 25(OH)D_3_, 25-hydroxyvitamin D_3_; SNP, single nucleotide polymorphism; NOS, Newcastle Ottawa scale*.

**Figure 2:**
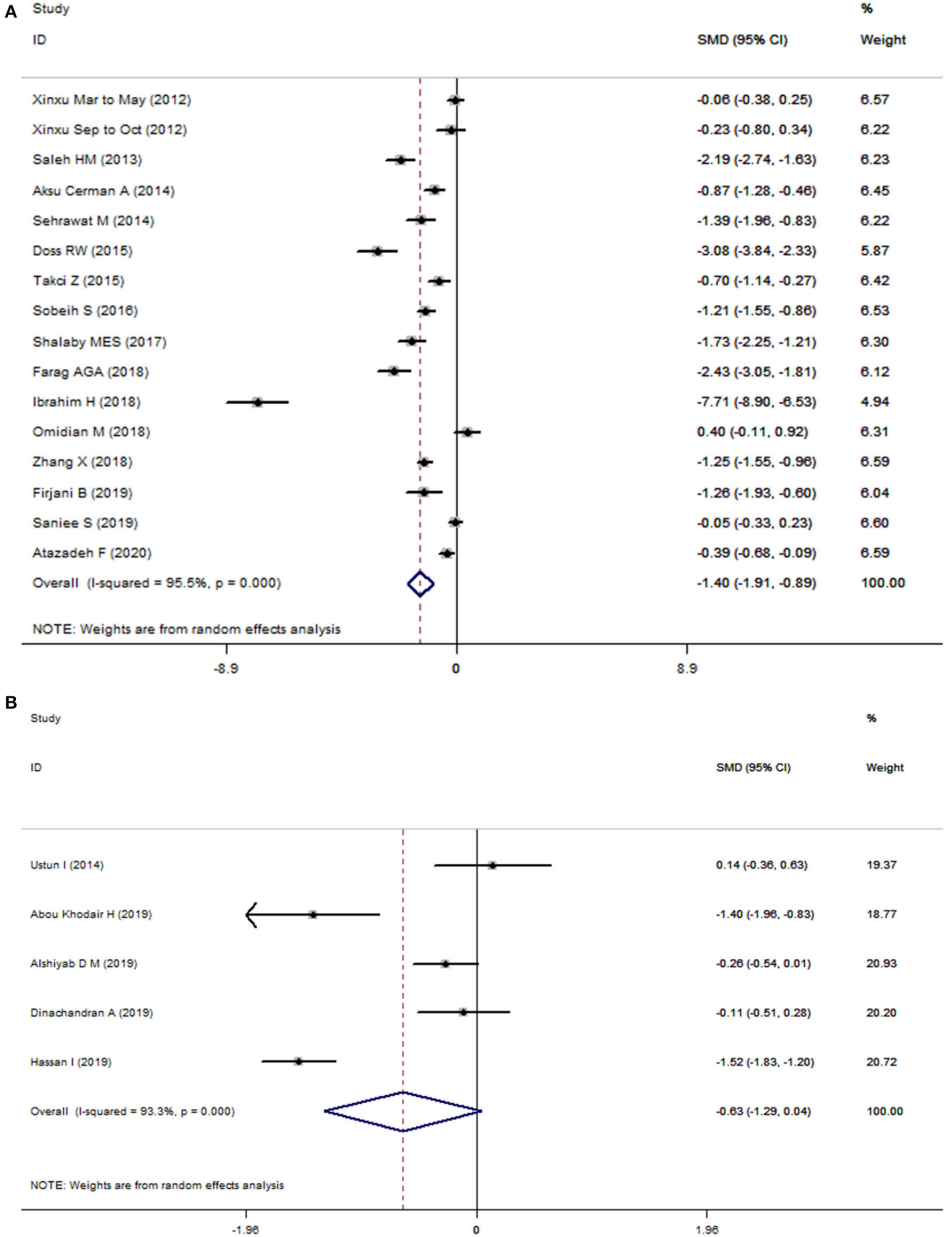
Forest plots of 25(OH)D **(A)** and 25(OH)D3 **(B)** in vitiligo patients relative to controls. The horizontal lines correspond to the study-specific standardized mean differences (SMD) and 95% confidence intervals (CI), respectively. The area of the squares reflects the study-specific weight.

Statistically significant heterogeneity was observed among studies for the association of 25(OH)D (*I*^2^ = 95.5%, *P* < 0.001) and 25(OH)D_3_ (*I*^2^ = 93.3%, *P* < 0.001) with risk of vitiligo, respectively. Therefore, we used random-effects models to combine the effect sizes. As shown in Figure 2 Patients with vitiligo had a lower level of 25(OH)D compared with controls (SMD = −1.40; 95% CI: −1.91, −0.89; *P* < 0.001). However, the differences of circulating 25(OH)D_3_ levels in cases and controls did not reach statistical significance (SMD = −0.63; 95% CI: −1.29, 0.04; *P* = 0.064).

Consistently, subgroup analysis revealed associations between circulating levels of 25(OH)D and risk of vitiligo in different strata. The levels of 25(OH)D_3_ in patients with vitiligo was significantly lower than that of the control group in the subgroups of studies with a high-quality score (SMD = −0.81; 95% CI: −1.56, −0.07; *P* = 0.032), published in 2017 and before (SMD = −0.81; 95% CI: −1.56, −0.07; *P* = 0.032), and in Asian populations (SMD = −1.40; 95% CI: −1.96, −0.83; *P* < 0.001). However, meta-regression analysis did not find potential sources of heterogeneity from region, publication year, quality score, study type and sample size for 25(OH)D and 25(OH)D_3_ (Supplementary Table 2). The Begg's test (*P* = 0.010) and Egger's test (*P* = 0.003) suggest evidence of potential publication bias in 25(OH)D, and the funnel plot was not symmetrical (Supplementary Figure 1A). For 25(OH)D_3_, the Begg's test (*P* = 0.806) and Egger's test (*P* = 0.966) did not suggest evidence of publication bias (Supplementary Figure 1B). Sensitivity analysis omitting one study at a time suggested none of these studies had a strong effect on the combined effect estimates (Supplementary Figure 2).

### Genetically Determined Circulating 25(OH)D and 25(OH)D_3_ Were Not Associated With Risk of Vitiligo

Supplementary Table 3 presents detailed information of IVs and their effect estimates with 25(OH)D, 25(OH)D_3_ and vitiligo, respectively. The F-statistic was 495.99 for 25(OH)D and 278.07 for 25(OH)D_3_, suggesting the IVs were unlikely to suffer from weak instrument bias.

As shown in Table 2, genetically predicted circulating 25(OH)D (OR = 0.93, 95% CI = 0.66–1.31, *P* = 0.663) and 25(OH)D_3_ levels (OR = 0.95, 95% CI = 0.80–1.14, *P* = 0.595) were not associated with altered risk of vitiligo using the IVW method. Similar results were obtained using the weighted-median approach and maximum-likelihood method. The intercept from the MR-Egger regression analysis did not reach statistical significance [25(OH)D*: P* = 0.614, 25(OH)D_3_: *P* = 0.061], suggesting no apparent evidence of directional pleiotropy. Additionally, no outlier SNPs were detected using MR-PRESSO test, and the causal effect estimates of 25(OH)D (*P* = 0.529) and 25(OH)D_3_ (*P* = 0.215) with risk of vitiligo were not statistically significant.

**Table 2:** Association of genetically predicted circulating levels of 25(OH)D and 25(OH)D_3_ levels with risk of Vitiligo.

	**No. of SNPs**	**OR (95% CI)**	***P* for association**	***P* for heterogeneity**	***P* intercept from MR-Egger regression**	***P* for MR-PRESSO global test**
**25(OH)D**
Inverse-variance weighted	10	0.93 (0.66–1.31)	0.667	0.419		
Weighted-median	10	0.90 (0.60–1.36)	0.622			
Maximum-likelihood	10	0.93 (0.66–1.31)	0.667			
MR-PRESSO test	10	0.93 (0.66–1.31)	0.677			0.529
MR-Egger	10	0.85 (0.53–1.39)	0.520		0.614	
**25(OH)D**_**3**_
Inverse-variance weighted	7	0.95(0.80–1.14)	0.595	0.326		
Weighted-median	7	0.91(0.75–1.11)	0.337			
Maximum-likelihood	7	0.95 (0.79–1.16)	0.620			
MR-PRESSO test	7	0.95 (0.79–1.15)	0.639			0.215
MR-Egger	7	0.75 (0.55–1.02)	0.066		0.061	

*25(OH)D, 25-hydroxyvitamin D; 25(OH)D_3_, 25-hydroxyvitamin D_3_; CI, confidence interval; MR, Mendelian randomization; MR-PRESSO test, MR-Pleiotropy RESidual Sum and Outlier test; OR, odds ratio; SNP, single nucleotide polymorphism*.

In the sensitivity analyses, we performed “leave-one-out” analyses to identify potential influencing SNPs. We found that the risk estimates of genetically predicted 25(OH)D and 25(OH)D_3_ with risk of vitiligo did not change substantially after excluding one single SNP at a time (Supplementary Figure 3). Then we excluded potential pleiotropic SNPs and used the remaining seven and five SNPs as IVs for 25(OH)D and 25(OH)D_3_. Details of the excluded SNPs and their associated phenotypes according to the GWAS Catalog are shown in Supplementary Table 4. Consistently, we did not observe statistically significant association of genetically predicted circulating levels of 25(OH)D (OR = 0.98, 95% CI = 0.63–1.53, *P* = 0.920) or 25(OH)D_3_ (OR = 1.16, 95% CI = 0.85–1.58, *P* = 0.360) with risk of vitiligo (Supplementary Table 5).

## Discussion

To date, vitiligo is a disease that cannot be completely cured. Patients often encounter psychological difficulties due to discrimination, such as shame, depression and anxiety, which usually lead to inferiority and social isolation (47, 48), and have an adverse impact on the quality of life (49). At present, the pathogenic mechanism of vitiligo is not completely clear. There are studies suggesting that vitamin D may increase the melanogenesis and tyrosinase content of human melanocytes through its anti-apoptotic effect, thus preventing the loss of skin pigment (50). In addition, vitamin D can also inhibit the autoimmune pathway in the pathogenesis of vitiligo by inhibiting the expression of interleukin-6 (IL-6), interleukin-8 (IL-8), tumor necrosis factor α (TNF-α) and tumor necrosis factor γ (TNF-γ), regulating the mature differentiation and activation of dendritic cells and inhibiting antigen presentation (51). For prevention and early diagnosis of vitiligo, the present study investigated the role of circulating 25(OH)D and 25(OH)D_3_ on the risk of vitiligo by using meta-analysis and Mendelian randomization, however, we did not find convincing evidence to support a protective role of vitamin D supplementation on the risk of vitiligo.

The results from the meta-analysis indicated that patients with vitiligo had a lower level of 25(OH)D compared with controls. Consistently, a meta-analysis conducted by Zhang et al. (52) in 2018 including 679 patients and 537 controls reported similar results (SMD = −0.94, 95% CI: −1.39, −0.48, *P* = 0.0001). Our updated meta-analysis included five additional recent published studies with a sample size of 987 patients and 770 controls for 25(OH)D, 305 patients and 321 controls for 25(OH)D_3_, and therefore had increased statistical power. Similarly, we also found heterogeneity in the studies included in the meta-analysis. However, meta-regression did not suggest potential sources of heterogeneity, and subgroup analysis consistently revealed associations between circulating levels of 25(OH)D and risk of vitiligo in different strata. Publication bias was detected for the association between 25(OH)D and risk of vitiligo, suggesting that our results may be more based on positive results, resulting in overestimation of the summary effect. Previously, no meta-analysis has focused on the association between 25(OH)D_3_ and risk of vitiligo. In the present study, we found no statistical differences in circulating levels of 25(OH)D_3_ between cases and controls. However, the number of included studies was relatively small, more studies are warranted for obtaining stable results. Additionally, owing to the limitations inherent in observational studies, high heterogeneity among studies may be due to differences in the study design, characteristics of the study population such as age and ethnicity, use of drugs and so on. Finally, all of the included studies were cross-sectional studies and case-control studies with relatively small study sample sizes, which is weak in demonstrating causality, since it is difficult to judge the time sequence of exposure and disease (53). Therefore, low vitamin D levels could also be a consequence of already developed vitiligo.

In order to overcome the limitations inherent in conventional observational studies, we adopted a two-sample MR method which used genetic variants as IVs of exposures to further investigate the relationship of 25(OH)D and 25(OH)D_3_ levels with risk of vitiligo. MR approach offers great opportunities to the etiological research of diseases, provided that the following three assumptions are satisfied (54). The first assumption is that the IVs must be associated with the exposure of interest. To ensure this, we used independent loci associated with circulating 25(OH)D and 25(OH)D_3_ levels achieving genome-wide significance level (*P* < 5 × 10^−8^) as IVs, which was identified from the largest GWAS to date. The second assumption is that the IVs must not be associated with potential confounders of the exposure-outcome association. Since genotypes are randomly allocated during gamete formation, MR analyses using genetic variants as IVs to a large extent solve the problem of confounding in conventional observational studies. The third assumption is that the IVs influence the outcome only through the risk factor. To ensure this, we excluded potential pleiotropic SNPs and retained those solely associated with 25(OH)D and 25(OH)D_3_ levels in the MR sensitivity analyses to evaluate the robustness of our results. We also performed different MR methods to test for potential pleiotropy. We did not observe evidence of directional pleiotropy for the causal association between 25(OH)D, 25(OH)D_3_ and risk of vitiligo in any of the above MR approaches. However, the estimate of MR studies reflect the effects of lifelong interference, whereas that of observational studies reflects more acute effects.

The limitations of the present study should be noted. First, the data for the meta-analysis were from observational studies, in which there could be potential confounding factors in the baseline characteristics of the selected population. The statistical ability of the small sample is limited, thus the well-designed and large sample studies are necessary to validate the findings. Second, heterogeneity in meta-analysis is a potential problem that affects the results of statistical analysis. In the present study, subgroup analysis and meta-regression did not indicate sources account for the heterogeneity observed in the study. In addition, publication bias was found in the results of 25(OH)D, which indicated that the results of meta-analysis were affected by publication bias. Third, the variance explained by IVs is limited, with 3.95% total 25(OH)D and 4.58% 25(OH)D_3_, respectively. Besides, the number of reported cases in the outcome dataset is relatively small, which could affect the statistical power in MR study. However, it is the largest sample size in the outcome database available at present, which needs more vitiligo GWASs with larger sample size to explore the association. Finally, because our MR analyses were restricted to participants of European ancestry, it is unclear whether our findings can be extrapolated to other study populations. Therefore, further prospective studies and functional studies *in vivo* and *in vitro* are needed to elucidate the exact role of 25(OH)D and 25(OH)D_3_ in the occurrence of vitiligo.

## Conclusions

Our study showed that although there seemed to be an inverse association between vitiligo and 25(OH)D level based on meta-analysis of observational studies, MR analysis pointed to a lack of causal association. In addition, meta-analysis and MR study did not provide evidence of an associations between 25(OH)D_3_ level and risk of vitiligo. The findings suggested there is no convincing evidence that vitamin D may help to prevent vitiligo.

## Data Availability Statement

The original contributions presented in the study are included in the article/Supplementary Material, further inquiries can be directed to the corresponding author/s.

## Author Contributions

JS performed the literature review, conducted data analysis, interpreted findings, and drafted the manuscript. KL and WC carried on data analysis and interpreted findings of meta-analysis. BL and HY mainly conducted on the data collation of the Mendelian randomization study. JS, LL, and XS can take responsibility for statistical reports, tables, and figures of the data analysis. YM and DY directed analytic strategy, supervised the study from conception to completion and revised drafts of the manuscript. All authors have read and approved the final manuscript.

## Funding

This work was supported by grants from the National Natural Science Foundation of China (81973663), the Natural Science Foundation of Zhejiang Province (LQ21H260001 and LQ20H260008), the Talent Project of Zhejiang Association for Science and Technology (2018YCGC003), the Foundation of Zhejiang Chinese Medical University (2020ZG01 and 2020ZG16), the Research Project of Zhejiang Chinese Medical University (2021JKZKTS004A).

## Conflict of Interest

The authors declare that the research was conducted in the absence of any commercial or financial relationships that could be construed as a potential conflict of interest.

## Publisher's Note

All claims expressed in this article are solely those of the authors and do not necessarily represent those of their affiliated organizations, or those of the publisher, the editors and the reviewers. Any product that may be evaluated in this article, or claim that may be made by its manufacturer, is not guaranteed or endorsed by the publisher.

## Abbreviations

CI: confidence interval
GWAS: genome-wide association study
IV: instrumental variable
IVW: inverse-variance weighted
IL-6: interleukin-6
IL-8: interleukin-8
MR: mendelian randomization
MAF: minor allele frequency
NOS: newcastle ottawa scale
OR: odds ratio
PRESSO: Pleiotropy RESidual Sum and Outlier
SD: standard deviation
SMD: standardized mean difference
SNP: single nucleotide polymorphism
TNF-α: tumor necrosis factor α
TNF-γ: tumor necrosis factor γ
25(OH)D: 25-hydroxyvitamin D
25(OH)D_3_: 25-hydroxyvitamin D_3_.
