# Supplementary material for: Circulating Vitamin D Levels and Risk of Vitiligo: Evidence From Meta-Analysis and Two-Sample Mendelian Randomization
Source: Front Nutr. 2021 Dec 22;8:782270. doi: 10.3389/fnut.2021.782270 (PMC8727691; doi:10.3389/fnut.2021.782270)
Supplement: Supplementary file 1 [file Data_Sheet_1.docx]

Supplementary materials

**Supplementary Table 1**. Detailed information of studies and datasets used in the present study.

**Supplementary Table 2**. Subgroup analysis for the association of 25(OH)D and 25(OH)D_3_ with the risk of vitiligo.

**Supplementary Table 3**. Associations of the instrumental SNPs with circulating levels of 25(OH)D, 25(OH)D_3_ and the risk of vitiligo.

**Supplementary Table 4**. Potential pleiotropic associations of the SNPs used as IVs for 25(OH)D and 25(OH)D_3_.

**Supplementary Table 5**. MR effect estimates of 25(OH)D and 25(OH)D_3_ with the risk of vitiligo from sensitivity analysis after removing potential pleiotropic SNPs.

**Figure S1.** Funnel plots for the associations of 25(OH)D and 25(OH)D_3_ with the risk of vitiligo. Each point represents a separate study. (**a)** 25(OH)D; (**b)** 25(OH)D_3_. The horizontal and vertical axis correspond to the SMD and comfidence limits. (SMD, standardized mean differences; se, standard error).

Abbreviations: 25(OH)D, 25-hydroxyvitamin D; 25(OH)D_3_, 25-hydroxyvitamin D_3_; SMD, standardized mean differences; se, standard error

**Figure S2.** Plots for sensitivity analysis omitting one study at a time for the association of 25(OH)D (**a**) and 25(OH)D_3_ (**b**) with the risk of vitiligo.

Abbreviations: 25(OH)D, 25-hydroxyvitamin D; 25(OH)D_3_, 25-hydroxyvitamin D_3_

**Figure S3.** Leave-one-out sensitivity analysis for the association of genetically predicted 25(OH)D (**a**) and 25(OH)D_3_ (**b**) with the risk of vitiligo. The horizontal lines correspond to ORs and 95% CIs of MR method.

Abbreviations: 25(OH)D, 25-hydroxyvitamin D; 25(OH)D_3_, 25-hydroxyvitamin D_3_; SMD, standardized mean differences; CI, confidence interval; OR, odds ratio.

| **Supplementary Table 1. Detailed information of studies and datasets used in the present study.** | | | | | | | | |
| --- | --- | --- | --- | --- | --- | --- | --- | --- |
| Exposure or outcome | Study or consortium | Participants | Ancestry | Web source |  |  |  |  |
| 25(OH)D | Ju-Sheng Zheng et al | 120,618 individuals | European ancestry | <https://doi.org/10.1371/journal.pmed.1003394> |  |  |  |  |
| 25(OH)D_3_ |  | 40,562 individuals | European ancestry |  |  |  |  |  |
| Vitiligo | Ying Jin et al | 4,680 cases and 39,586 controls | European ancestry | <https://www.nature.com/articles/ng.3680> |  |  |  |  |
|  |  |  |  |  |  |  |  |  |
|  |  |  |  |  |  |  |  |  |

Abbreviations: 25(OH)D, 25-hydroxyvitamin D; 25(OH)D_3_, 25-hydroxyvitamin D_3_

**Supplementary Table 2. Subgroup analysis for the association of 25(OH)D and 25(OH)D_3_ with the risk of vitiligo.**

|  |  | **25(OH)D** | | | | |  | **25(OH)D_3_** | | | | |
| --- | --- | --- | --- | --- | --- | --- | --- | --- | --- | --- | --- | --- |
|  | Type | Number of documents | SMD (95%*CI*) | *P-*value | *I*2 *(%)* | *P-*value of interlayer heterogeneity |  | Number of documents | SMD (95%CI) | *P-value* | *I*2 (%) | *P*-value of interlayer heterogeneity |
|  |  |  |  |  |  |  |  |  |  |  |  |  |
|  |  |  |  |  |  |  |  |  |  |  |  |  |
| Total |  | 15 | -1.40(-1.91, -0.89) | <0.001 | 95.5 |  |  | 5 | -0.63(-1.29, 0.04) | 0.064 | 93.3 |  |
| publication year |  | | | | | | | | |  |  |  |
|  | <2017 | 7 | -1.18(-1.76, -0.60) | <0.001 | 92.4 | 0.586 |  | 4 | -0.81(-1.56, -0.07) | 0.032 | 93.9 | 0.341 |
|  | ≥2017 | 8 | -1.67(-2.55, -0.79) | <0.001 | 97.0 |  |  | 1 | 0.14(-0.36, 0.64) | 0.590 | / |  |
| study type |  | | | | | | | | |  |  |  |
|  | Case-control study | 14 | -1.45(-1.88, -0.84) | <0.001 | 95.8 | 0.746 |  | 4 | -0.81(-1.56, -0.07) | 0.032 | 93.9 | 0.341 |
|  | Cross sectional study | 1 | -0.87(-1.28, -0.46) | <0.001 | / |  |  | 1 | 0.14(-0.36, 0.63) | 0.590 | / |  |
| region |  | | | | | | | | |  |  |  |
|  | Europe | 1 | -0.87(-1.28, -0.46) | <0.001 | / | 0.095 |  | 0 | / |  |  |  |
|  | Africa | 7 | -2.70(-3.71, -1.69) | <0.001 | 95.2 |  |  | 1 | -1.40(-1.96, -0.83) | / | / | 0.351 |
|  | Asia | 7 | -0.46(-0.86, -0.05) | <0.001 | 89.4 |  |  | 4 | -0.45(-1.20, 0.29) | 0.235 | 94.3 |  |
| sample size |  | | | | | | | | |  |  |  |
|  | >100 | 7 | -1.31(-1.99, -0.64) | <0.001 | 96.5 | 0.980 |  | 3 | -0.64(-1.51, 0.24) | 0.156 | 95.4 | 0.987 |
|  | ≤100 | 8 | -1.48(-2.27, -0.68) | <0.001 | 93.2 |  |  | 2 | -0.62(-2.13, 0.88) | 0.416 | 93.7 |  |
| quality score |  | | | | | | | | |  |  |  |
|  | >7 | 4 | -1.83(-2.38, -1.27) | <0.001 | 70.8 | 0.680 |  | 4 | -0.81(-1.56, -0.07) | 0.032 | 93.9 | 0.341 |
|  | ≤7 | 11 | -1.26(-1.84, -0.68) | <0.001 | 96.0 |  |  | 1 | 0.14(-0.36, 0.64) | 0.590 | / |  |

Abbreviations: 25(OH)D, 25-hydroxyvitamin D; 25(OH)D_3_, 25-hydroxyvitamin D_3_; SMD, standardized mean differences; 95%CI, 95% confidence intervals

**Supplementary Table 3. Associations of the instrumental SNPs with circulating levels of 25(OH)D, 25(OH)D_3_ and the risk of vitiligo.**

| SNP | | Chr | | Position | | EA/NEA | SNP-Exposure | | | | | | SNP-Outcome | | | | |
| --- | --- | --- | --- | --- | --- | --- | --- | --- | --- | --- | --- | --- | --- | --- | --- | --- | --- |
|  |  |  |  |  |  |  | β | | SE | | *P*-value | | | β | SE | | *P*-value |
| **25(OH)D** | |  | |  | |  |  | |  | |  | | |  |  | |  |
| rs11203339 | | 1 | | 17560972 | T/C | | 0.012 | | 0.002 | | 4.64× 10^-08^ | | 0.020 | | 0.030 | 0.505 |  |
| rs116970203 | | 11 | | 14876718 | A/G | | 0.381 | | 0.022 | | 1.19× 10^-64^ | | 0.010 | | 0.100 | 0.950 |  |
| rs12785878 | | 11 | | 71167449 | G/T | | 0.044 | | 0.002 | | 5.60× 10^-87^ | | -0.010 | | 0.030 | 0.745 |  |
| rs17216707 | | 20 | | 52732362 | C/T | | 0.030 | | 0.003 | | 1.61× 10^-29^ | | 0.000 | | 0.040 | 0.931 |  |
| rs17862870 | | 2 | | 234622742 | A/G | | 0.021 | | 0.004 | | 5.57× 10^-09^ | | -0.020 | | 0.050 | 0.631 |  |
| rs3213737 | | 12 | | 96379806 | G/A | | 0.019 | | 0.002 | | 2.05× 10^-19^ | | 0.068 | | 0.030 | 0.027 |  |
| rs3755967 | | 4 | | 72609398 | T/C | | 0.106 | | 0.002 | | 2.48× 10^-465^ | | 0.030 | | 0.030 | 0.281 |  |
| rs7529325 | | 1 | | 152492634 | A/T | | 0.030 | | 0.005 | | 2.09× 10^-09^ | | -0.073 | | 0.050 | 0.202 |  |
| rs8018720 | | 14 | | 39556185 | G/C | | 0.018 | | 0.003 | | 1.46× 10^-10^ | | -0.020 | | 0.040 | 0.598 |  |
| rs9304669 | | 19 | | 48384385 | T/C | | 0.052 | | 0.01 | | 4.53× 10^-08^ | | 0.020 | | 0.040 | 0.547 |  |
| **25(OH)D_3_** | |  | |  | |  |  | |  | |  | | |  |  | |  |
| rs116970203 | | 11 | | 14876718 | | C/A | 0.055 | | 0.010 | | 1.94×10-8 | | | 1.010 | 0.100 | | 0.505 |
| rs13084927 | | 3 | | 72709792 | | G/T | 0.266 | | 0.008 | | 6.55×10-266 | | | 1.010 | 0.040 | | 0.950 |
| rs17216707 | | 20 | | 52732362 | | G/A | 0.372 | | 0.023 | | 3.22×10-60 | | | 1.000 | 0.040 | | 0.745 |
| rs28364617 | | 12 | | 71159764 | | G/T | 0.127 | | 0.008 | | 4.08×10-59 | | | 0.990 | 0.030 | | 0.931 |
| rs3819817 | | 11 | | 96378771 | | C/T | 0.058 | | 0.007 | | 3.59×10-16 | | | 1.070 | 0.030 | | 0.631 |
| rs4588 | | 4 | | 72618323 | | T/C | 0.054 | | 0.010 | | 1.27×10-08 | | | 1.040 | 0.030 | | 0.027 |
| rs9304669 | | 19 | | 48384385 | | T/C | 0.074 | | 0.009 | | 1.09×10-15 | | | 1.020 | 0.040 | | 0.281 |
| Abbreviations: 25(OH)D, 25-hydroxyvitamin D; 25(OH)D_3_, 25-hydroxyvitamin D_3_; Chr, chromosome; EA, effect allele; NEA, non-effect allele; SE, standard error; SNP, single nucleotide polymorphism. | | | | | | | | | | | | | | | | | |

| **Supplementary Table 4. Potential pleiotropic associations of the SNPs used as IVs for 25(OH)D and 25(OH)D_3_.** | | | | | | | | | | | | |
| --- | --- | --- | --- | --- | --- | --- | --- | --- | --- | --- | --- | --- |
| SNP | | Trait(s) | | *P*-value | | PubMed ID | | Location | |  |  |  |
| **25(OH)D** | |  | |  | |  | |  | |  |  |  |
| rs7529325 | | NA | | NA | | NA | | NA | |  |  |  |
| rs11203339 | | NA | | NA | | NA | | NA | |  |  |  |
| rs3755967 | | Leukocyte count | | 4×10^-32^ | | 30595370 | | 4:71743681 | |  |  |  |
| rs3213737 | | Chronic kidney disease | | 2×10^-25^ | | 31959995 | | 12:95986028 | |  |  |  |
|  | | Keratinocyte carcinoma | | 1×10^-8^ | | 31174203 | | 12:95986028 | |  |  |  |
|  | | Sunburn | | 3×10^-48^ | | 30595370 | | 12:95986028 | |  |  |  |
| rs8018720 | | NA | | NA | | NA | | NA | |  |  |  |
| rs116970203 | | NA | | NA | | NA | | NA | |  |  |  |
| rs8018720 | | NA | | NA | | NA | | NA | |  |  |  |
| rs12785878 | | NA | | NA | | NA | | NA | |  |  |  |
| rs17216707 | | Nephrolithiasis | | 1×10^-11^ | | 31729369 | | 20:54115823 | |  |  |  |
|  | | Nephrolithiasis | | 8×10^-18^ | | 31729369 | | 20:54115823 | |  |  |  |
|  | | Creatinine measurement | | 9×10^-15^ | | 26831199 | | 20:54115823 | |  |  |  |
|  | | Calcium measurement | | 1×10^-8^ | | 31993563 | | 20:54115823 | |  |  |  |
|  | | Glomerular filtration rate | | 2×10^-46^ | | 31152163 | | 20:54115823 | |  |  |  |
|  | | Fibroblast growth factor 23 measurement | | 3×10^-24^ | | 30217807 | | 20:54115823 | |  |  |  |
|  | | Alkaline phosphatase measurement | | 7×10^-9^ | | 33547301 | | 20:54115823 | |  |  |  |
|  | | Glomerular filtration rate | | 1×10^-17^ | | 31015462 | | 20:54115823 | |  |  |  |
|  | | Glomerular filtration rate | | 5×10^-16^ | | 30604766 | | 20:54115823 | |  |  |  |
| rs9304669 | | NA | | NA | | NA | | NA | |  |  |  |
| **25(OH)D_3_** | | | | | | | | | |  |  |  |
| rs3819817 | | NA | | NA | | NA | | NA | |  |  |  |
| rs116970203 | | NA | | NA | | NA | | NA | |  |  |  |
| rs13084927 | | NA | | NA | | NA | | NA | |  |  |  |
| rs28364617 | | NA | | NA | | NA | | NA | |  |  |  |
| rs17216707 | | Nephrolithiasis | | 1×10^-11^ | | 31729369 | | 20:54115823 | |  |  |  |
|  | | Nephrolithiasis | | 8×10^-18^ | | 31729369 | | 20:54115823 | |  |  |  |
|  | | Creatinine measurement | | 9×10^-15^ | | 26831199 | | 20:54115823 | |  |  |  |
|  | | Calcium measurement | | 1×10^-8^ | | 31993563 | | 20:54115823 | |  |  |  |
|  | | Glomerular filtration rate | | 2×10^-46^ | | 31152163 | | 20:54115823 | |  |  |  |
|  | | Fibroblast growth factor 23 measurement | | 3×10^-24^ | | 30217807 | | 20:54115823 | |  |  |  |
|  | | Alkaline phosphatase measurement | | 7×10^-9^ | | 33547301 | | 20:54115823 | |  |  |  |
|  | | Glomerular filtration rate | | 1×10^-17^ | | 31015462 | | 20:54115823 | |  |  |  |
|  | | Glomerular filtration rate | | 5×10^-16^ | | 30604766 | | 20:54115823 | |  |  |  |
| rs9304669 | | NA | | NA | | NA | | NA | |  |  |  |
| rs4588 | | Myeloid white cell count | | 2×10^-48^ | | 32888493 | | 4:71752606 | |  |  |  |
|  | | Platelet crit | | 2×10^-11^ | | 32888494 | | 4:71752606 | |  |  |  |
|  | | Platelet count | | 4×10^-9^ | | 32888494 | | 4:71752606 | |  |  |  |

Abbreviations: IV, instrumental variables; SNP, single nucleotide polymorphism

**Supplementary Table 5. MR effect estimates of 25(OH)D and 25(OH)D_3_ with the risk of vitiligo from sensitivity analysis after removing potential pleiotropic SNPs.**

|  | No. of SNPs | OR (95% CI) | *P* for association | *P* for heterogeneity | *P* intercept from MR-Egger regression | *P* for MR-PRESSO Global test |
| --- | --- | --- | --- | --- | --- | --- |
| 25(OH)D |  |  |  |  |  |  |
| Inverse-variance weighted | 7 | 0.98 (0.63-1.53) | 0.921 | 0.768 |  |  |
| Weighted-median | 7 | 1.02 (0.63-1.68) | 0.923 |  |  |  |
| Maximum-likelihood | 7 | 0.98 (0.63-1.53) | 0.920 |  |  |  |
| MR-PRESSO test | 7 | 0.98 (0.70-1.36) | 0.898 |  |  | 0.862 |
| MR-Egger | 7 | 1.06 (0.62-1.83 | 0.824 |  | 0.591 |  |
| 25(OH)D_3_ |  |  |  |  |  |  |
| Inverse-variance weighted | 5 | 1.16(0.85-1.58) | 0.360 | 0.322 |  |  |
| Weighted-median | 5 | 1.05(0.73-1.49) | 0.802 |  |  |  |
| Maximum-likelihood | 5 | 1.16 (0.83-1.63) | 0.395 |  |  |  |
| MR-PRESSO test | 5 | 1.16 (0.83-1.62) | 0.445 |  |  | 0.443 |
| MR-Egger | 5 | 0.83 (0.47-1.46) | 0.523 |  | 0.167 |  |

Abbreviations: SNP, single nucleotide polymorphism; 25(OH)D, 25-hydroxyvitamin D; 25(OH)D_3_, 25-hydroxyvitamin D_3_; CI, confidence interval; MR, Mendelian randomization; MR-PRESSO test, MR-Pleiotropy RESidual Sum and Outlier test; OR, odds ratio.


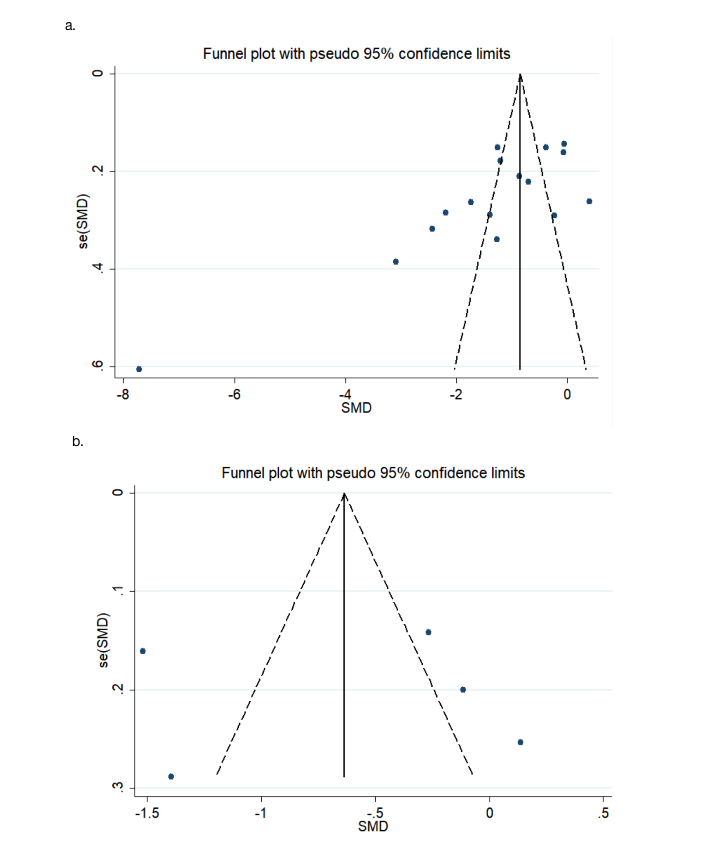


**Figure S1**. Funnel plots for the associations of 25(OH)D and 25(OH)D_3_ with the risk of vitiligo. Each point represents a separate study. (**a)** 25(OH)D; (**b)** 25(OH)D_3_. The horizontal and vertical axis corresponds to SMDs and confidence limits. (SMD, standardized mean differences; se, standard error).


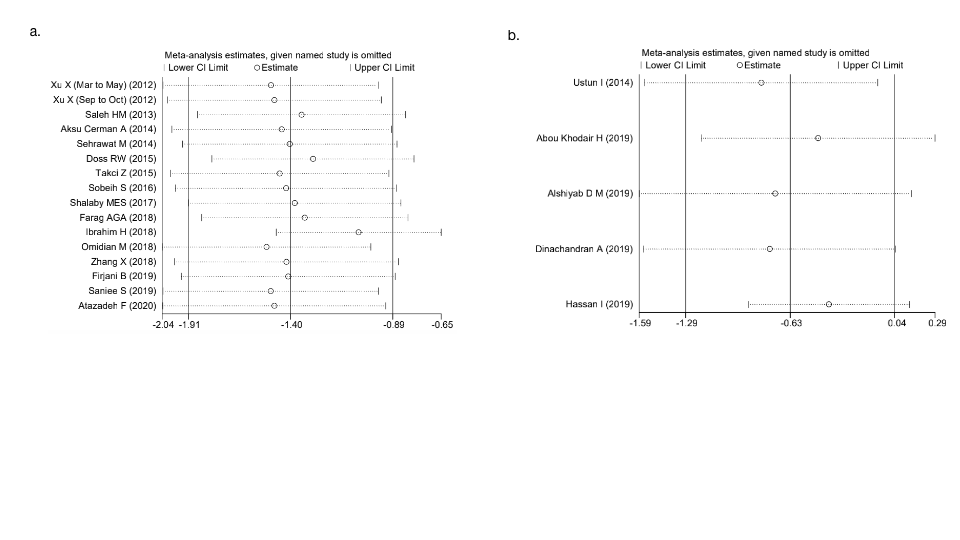


**Figure S2**. Plots for sensitivity analysis omitting one study at a time for the associations of 25(OH)D (**a**) and 25(OH)D_3_ (**b**) with the risk of vitiligo.


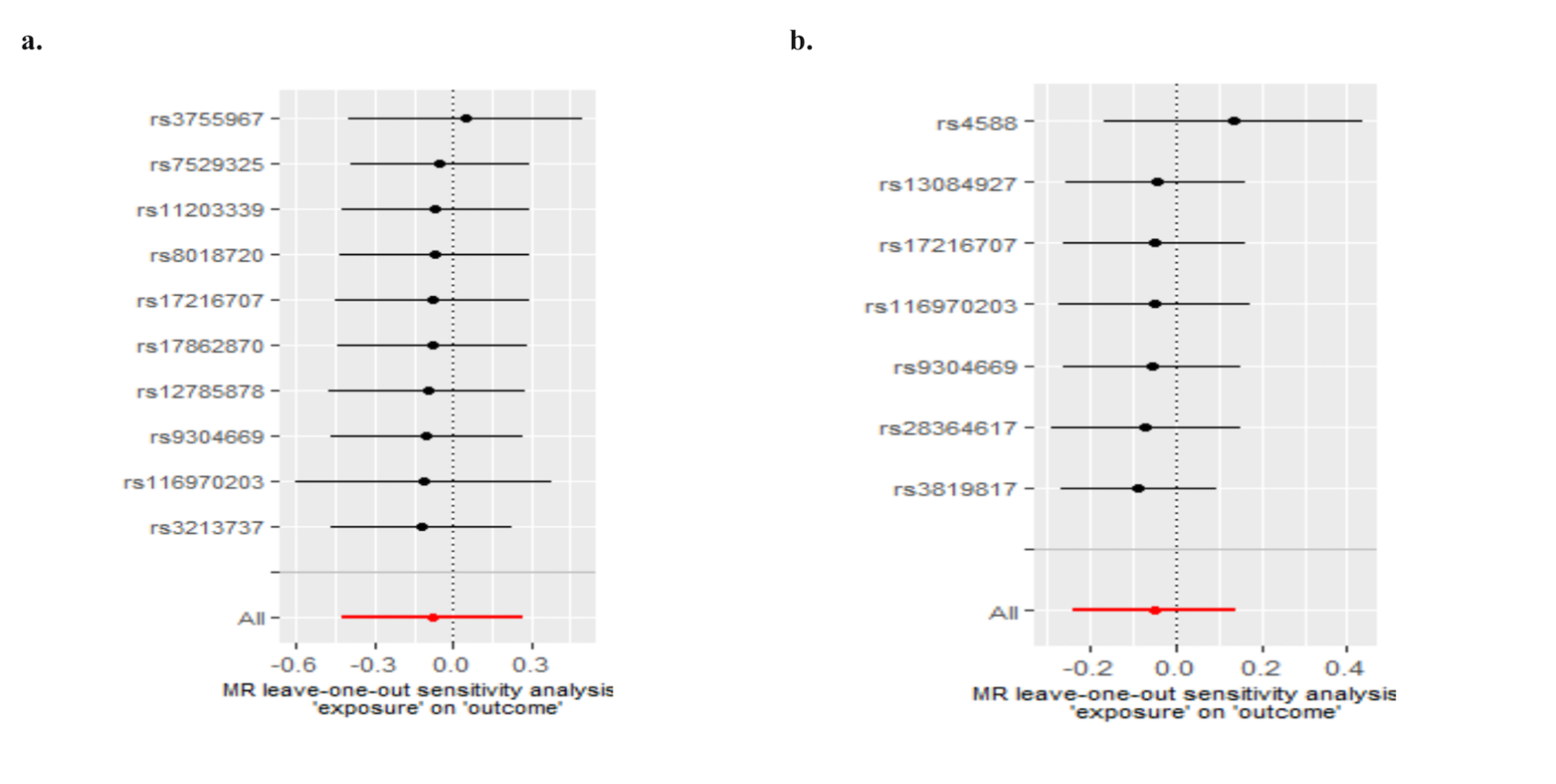


**Figure S3**. Leave-one-out sensitivity analysis for the association of genetically predicted 25(OH)D (**a**) and 25(OH)D_3_ (**b**) with the risk of vitiligo. The horizontal lines correspond to ORs and 95% CIs of MR method. CI, confidence interval; OR, odds ratio.
